# The roles of ERIANIN in tumor and innate immunity and its’ perspectives in immunotherapy

**DOI:** 10.3389/fimmu.2023.1170754

**Published:** 2023-04-28

**Authors:** Zhen Yang, Ruxue Liu, Minghan Qiu, Hanwei Mei, Jie Hao, Teng Song, Ke Zhao, Dandan Zou, Huaqing Wang, Ming Gao

**Affiliations:** ^1^ Department of Oncology, Tianjin Union Medical Center of Nankai University, Tianjin, China; ^2^ The Institute of Translational Medicine, Tianjin Union Medical Center of Nankai University, Tianjin, China; ^3^ College of Integrative Medicine, Tianjin University of Traditional Chinese Medicine, Tianjin, China; ^4^ Department of Thyroid and Breast Surgery, Tianjin Key Laboratory of General Surgery in Construction, Tianjin Union Medical Center, Tianjin, China

**Keywords:** traditional Chinese medicine, ERIANIN, cancer, innate immunity, signaling pathways, immunotherapy

## Abstract

Traditional Chinese medicine has been used in China for thousands of years. In 2022, the 14th Five-Year Plan for the Development of Traditional Chinese Medicine was released, aiming to enhance traditional Chinese medicine health services and improve policies and systems for high-quality traditional Chinese medicinal development by 2025. ERIANIN, the main component of the traditional Chinese medicine Dendrobium, plays an important role in anti-inflammatory, antiviral, antitumor, antiangiogenic, and other pharmacological effects. ERIANIN has broad-spectrum antitumor effects, and its tumor-suppressive effects have been confirmed in the study of various diseases, such as precancerous lesions of the stomach, gastric cancer, liver cancer, lung cancer, prostate cancer, bladder cancer, breast cancer, cervical cancer, osteosarcoma, colorectal cancer, leukaemia, nasopharyngeal cancer and melanoma through the multiple signaling pathways. Thus, the aim of this review was to systematically summarise the research on ERIANIN with the aim of serving as a reference for future research on this compound and briefly discuss some future perspectives development of ERIANIN in combined immunotherapy.

## 1 Background

Natural products have had a longstanding role in drug discovery and development, and the unique biological activities of these products are continuously emerging (1–3). Natural products and their derivatives, which are characterised by structural diversity and high biological activity, have exhibited a wide range of pharmacological activities in the treatment of common clinical diseases, such as heart diseases, infectious diseases, skin diseases, especially in malignant tumors (4–6). The global tumor burden continues to increase, and malignant tumors with biological features such as abnormal cell differentiation, proliferation, metastasis and the suppressive immune microenvironments have become a worldwide problem and a serious threat to human health (7, 8). In addition, drug resistance in malignant cells is the primary cause of cancer treatment failure, and the side effects and high cost of cancer therapies limit their clinical application (9–11). The active ingredients of traditional Chinese medicine have been shown to enhance the body’s immune function, remodel the tumor microenvironment, and induce death of tumor cells, while also enhancing the effectiveness, reducing the toxicity, and reversing multidrug resistance when used in combination with chemotherapy drugs (12–14). The antitumor effects of traditional Chinese medicine are mainly divided into two types: exerting a direct inhibitory effect on the growth of tumor cells and an indirect effect of enhancing damaged immune function so as to activate the immune response of tumor cells (15, 16).

ERIANIN, a natural biphenyl compound derived from the Chinese herbal medicine Dendrobium, is one of the most prominent chemical components and has been used as an antipyretic and analgesic agent (17). ERIANIN also plays an important role in the treatment of inflammation, diabetic nephropathy, retinopathy, psoriasis, and cancers including stomach, gastric cancer, liver cancer, lung cancer, prostate cancer, bladder cancer, breast cancer, cervical cancer, osteosarcoma, colorectal cancer, leukaemia, nasopharyngeal cancer and melanoma through the multiple signaling pathways and holds promise as a potential therapeutic agent for a variety of diseases (18, 19).For example, ERIANIN inhibited PDL1-mediated angiogenesis, proliferation, invasion and migration through the mTOR/p70S6K/4EBP1 pathway and RAS/Raf/MEK/MAPK-ERK pathway in cervical cancer (19).

In this review, we provide an overview of the role of ERIANIN, especially in cancer and innate immunity, and elaborates on its molecular mechanism related to anticancer activity through the well-known signalling pathways, including phosphoinositide 3-kinase (PI3K)/AKT pathway, MEK pathway, JNK pathway, NRF2/PLOOH pathways, Janus kinase (JAK)/signal transducer and activator of transcription 3 (STAT3) pathway, GSK3β pathway, and NLRP3/ROS pathway. Moreover, we discuss the potential value of ERIANIN in the treatment of diseases and to reveal its promise as a novel drug combined with immunotherapy for the prevention and treatment of cancer for clinical applications.

## 2 An overview of ERIANIN: structure, source and biological activity

**Figure 1:**
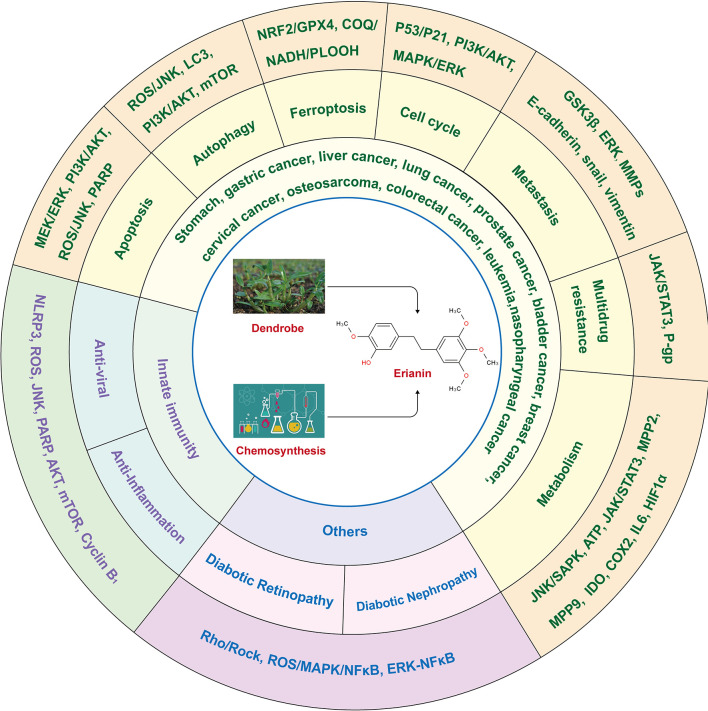
The structure and role of ERIANIN. The Chemdraw-Chem3D showed the secondary structure of ERIANIN. ERIANIN play a role in anti-tumor, anti-viral, anti-inflammation and the others pharmacological function by regulating the different biological phenotypes through the indicated pathways.

ERIANIN (C_18_H_22_O_5_) is a natural compound with a low molecular weight and is mainly isolated from Dendrobium officinale and Dendrobium chrysotoxum (17). Dendrobium is a traditional Chinese medicinal herb and a second-class protected plant in China, with rich medicinal value and a history in medicine of more than 2000 years (20). The genus Dendrobium includes Dendrobium officinale, Dendrobium huoshanense, Dendrobium nobile, and other species (21). It benefits the stomach, nourishes the yin, and clears heat. The main active ingredients of Dendrobium are alkaloids, polysaccharides, phenanthrenes, bibenzyls, flavonoids, sterols, terpenes, and amino acids, among which alkaloids are the main pharmacologically active ingredients (22). ERIANIN is an important bioactive component that exerts medicinal value and belongs to the bibenzyl group of compounds, with the chemical name 2-methoxy-5-[2-(3,4,5-trimethoxy-phenyl)-ethyl]-phenol. In addition to natural sources, ERIANIN can also be chemically synthesised (23). Pharmacological studies have shown effects such as anti-inflammatory, antiviral, antitumor, antiangiogenic, and other pharmacological effects (**Figure 1**).

## 3 Significances of ERIANIN in cancers

ERIANIN has broad-spectrum antitumor effects, and its tumor-suppressive effects have been confirmed in the study of various diseases, such as precancerous lesions of the stomach (24), gastric cancer (25), liver cancer (26–28), lung cancer (29–31), prostate cancer (32), bladder cancer (33), breast cancer (34, 35), cervical cancer (36), osteosarcoma (37), colorectal cancer (38, 39), leukaemia (40–42), nasopharyngeal cancer (43), and melanoma (44). In recent years, several cytotoxicity experiments have demonstrated the inhibitory effect of ERIANIN on tumor cells. By summarising the 50% inhibitory concentration (IC_50_) values of the experiments, it was found that the inhibitory effect of ERIANIN on different tumor cells was different and was mostly dose-dependent. However, the IC_50_ of 28/31 cancer cell lines at the specified point in time was below 100 nM, which indicated that ERIANIN has high specificity to tumor cells and good antitumor activity. The specific values are listed in (**Table 1**).

**Table 1:** Inhibitory effect of ERIANIN on different tumor cell lines.

Tumor	Tumor cell lines	IC_50_ (nM)	Time (hr)	Reference
PLGC	MC	80.00	48	(25)
Gastric cancer	SGC7901	175.90	48	(26)
Liver cancer	HepG2	43.69	24	(27)
	SMMC-7721	81.02	24	(27)
	Huh7	37.40	48	(28)
	PLC/PRF/5	55.00	72	(29)
	HLE	28.60	72	(29)
	Huh1	25.90	72	(29)
	Bel-7402	21.50	72	(29)
	SUN739	17.70	72	(29)
Lung cancer	A549	52.64	48	(30)
	H1975	0.51	48	(31)
	H460	25.00	72	(32)
	H1299	50.00	72	(32)
Prostate cancer	LNCaP	26.50	48	(33)
Bladder tumor	EJ	65.04	48	(34)
	T24	45.90	48	(34)
Breast cancer	MDA-MB-231	70.96	24	(35)
	EFM-192a	78.58	24	(35)
	T47D	68.4	72	(36)
Cervical cancer	HeLa	8300.00	48	(37)
Osteosarcoma	143B	40.97	48	(38)
	MG63.2	44.26	48	(38)
Colorectal cancer	SW480	24.50	48	(39)
	HCT116	45.00	48	(39)
	Caco-2	2654.19	48	(40)
Leukemia	HL-60	38.00	24	(40)
	JurkaT	42.55	48	(43)
	K562	14.13	72	(42)
Nasopharyngeal carcinoma	NPC-039	80.00	48	(44)
	NPC-BM	80.00	48	(44)

### 3.1 Apoptosis effects

Apoptosis, also known as programmed cell death, is an active physiological suicidal behaviour that plays an important role in normal development, maintenance of homeostasis, formation of immune tolerance, and tumor surveillance in multicellular organisms (45). Apoptosis is genetically controlled and energy dependent. In tumors, cells lose their ability to undergo apoptosis, so inducing apoptosis in tumor cells can effectively inhibit cancer progression and achieve the goal of cancer treatment. There are four apoptotic pathways in eukaryotic cells, including the endogenous (mitochondrial-mediated), exogenous (death receptor-mediated), granzyme, and endoplasmic reticulum stress (ERS) pathways (46, 47). The first two are recognised as classical apoptotic pathways (48). The granzyme pathway is mediated by cytotoxic T lymphocytes, which generate perforin to act on the cell membrane and release granzyme A and granzyme B intracellularly, leading to apoptosis (49). In recent years, ERS has been found to induce apoptosis (50). The accumulation of unfolded proteins in the endoplasmic reticulum causes ERS and regulates the BCL-2 family and Ca^2+^ channels, which in turn promotes the mitochondrial outer membrane permeabilization (MOMP) process to induce apoptosis (51), therefore, the ERS pathway can be regarded as a complement to the mitochondrial apoptotic pathway. The apoptotic pathways are different, but all end up with the same executive signalling pathway, namely the caspase family activation cascade (52). ERIANIN can induce typical apoptotic phenomena, such as cell crumpling, chromosome condensation, changes in mitochondrial membrane potential, and apoptotic vesicles.

The caspase family belongs to cysteine proteases that play initiation and effector roles in apoptosis (52). According to the mode of action and function of the cascade reaction of apoptosis, caspase family members are divided into two categories: apoptosis initiation factor promoters, including Caspases-2, 8, 9, and 10, and apoptosis effect factor executors, including Caspases-3, 6, and 7 (53, 54). In normal cells, caspases exist as inactive zymogens. Upon stimulation by apoptotic signals, the promoter becomes an active caspase through self-cleavage, activating other downstream caspases and causing a cascade reaction. Finally, the activated executor cleaves key proteins associated with apoptosis, such as protein kinases (protein kinase B, protein kinase C, focal adhesion kinase, and Raf 1), nuclear lamins, cellular structural proteins, and enzymes related to DNA repair, thereby causing apoptosis (55). Caspase-3 is one of the most important apoptotic execution factors in the caspase family and is the key protein kinase and main effector of apoptosis. Most apoptosis triggers require the caspase-3-mediated signalling pathway to complete apoptosis (56). The DNA repair enzyme PARP is a cleavage substrate of caspase and can be used as a marker of apoptosis. In leukaemia HL-60 cells (40), breast cancer MDA-MB-231 cells (34), and colon cancer SW480 cells (57), ERIANIN can induce caspase cascade reactions and PARP degradation. Treatment of breast cancer MDA-MB-231 cells with 40, 80, and 160 nM of ERIANIN for 24 h exhibited significant pro-apoptotic effects in a dose-dependent manner. ERIANIN upregulates the expression of caspase-3, caspase-9, PARP, and cytochrome C (Cyt-C), and changes the ratio of BCL-2/BAX. Cyt-C is a specific protein located in the mitochondria, indicating that ERIANIN induces apoptosis in MDA-MB-231 cells by mediating the mitochondrial pathway and activating the caspase cascade reaction. In addition, DAPI staining clearly revealed chromosome condensation and breakage in the nucleus, which are typical morphological manifestations of apoptosis (34).

Studies have shown that PI3K and ERK signalling pathways are involved in the process of cell apoptosis (58, 59). The PI3K/AKT pathway is an important signal transduction pathway in a variety of malignancies, and the PI3K protein family is involved in regulating various cellular functions, such as cell proliferation, differentiation, apoptosis, and glucose transport (60). Ras is an upstream regulatory gene of the PI3K/AKT signalling pathway and is involved in regulating the biological behaviour of cells (61). Ras can produce PIP3 upon activation, and PIP3 acts as a secondary messenger to recruit AKT proteins to the cell membrane and activate AKT. Phosphorylated AKT induces a cellular cascade response that activates antiapoptotic proteins and inhibits the expression of pro-apoptotic proteins (62). MDM2 is a protein downstream of AKT (63). After activation, it is translocated from the cytoplasm to the nucleus and interacts with the suppressor gene p53, inhibiting p53 expression and leading to p53 degradation. In cervical cancer cells (36), ERIANIN activates p53, increases the expression of BAX and Caspase-3, inhibits the proliferation of cervical cancer HeLa cells, and promotes apoptosis. ERIANIN also decreased the expression of phosphorylated ERK1/2 and inhibited the ERK1/2 signalling pathway to mediate mitochondrial apoptosis. Moreover, ERIANIN can treat gastric precancerous lesions by modulating the HRAS/PI3K/AKT pathway, inhibiting MC activity, decreasing MDM2 expression, enhancing p53 activity, promoting early apoptosis of gastric cancer cells, and preventing cancer (24).

The regulation of the PI3K/AKT pathway by ERIANIN is not unique to gastric cancer. In triple-negative breast cancer, ERIANIN downregulates the expression of p-PI3K and p-AKT, confirming that inhibition of the PI3K/AKT pathway plays an important role in the regulation of breast cancer (34). In hepatocellular carcinoma studies, ERIANIN inhibited the activation of the PI3K/AKT pathway and induced apoptosis in hepatocellular carcinoma cells by inhibiting AKT phosphorylation and promoting the expression of the negative regulatory protein PTEN. At the same time, it also inhibits hepatocellular carcinoma cells through the p38 and ERK/MAPK pathway (64). ERK1/2 phosphorylation was also reduced in a dose-dependent manner in ERIANIN-treated nasopharyngeal carcinoma cells. ERIANIN may induce apoptosis in human nasopharyngeal carcinoma cells through the ERK pathway (43). In lung cancer studies, ERIANIN upregulated the expression of BAX, Caspase-3, and Caspase-9, downregulated the expression of BCL-2, p-PI3K, p-AKT, and p-mTOR, affected apoptosis and pathway-related proteins, induced early and late apoptosis in H1975 lung cancer cells by regulating the PI3K/AKT/mTOR pathway, and inhibited the growth of human lung cancer cells (30). This suggests that the PI3K/AKT signalling pathway may be an important mechanism by which ERIANIN exerts its antitumor function.

BAX and BCL-2 are important apoptosis regulatory proteins (65). BCL-2 is located on the outer mitochondrial membrane and exerts antiapoptotic effects by inhibiting the permeability of the mitochondrial membrane and preventing the release of Cyt-C and inducible factors, thereby preventing caspase activation (66). BAX increases the permeability of the mitochondrial outer membrane, establishes outer membrane channels, promotes the release of Cyt-C and reactive oxygen species (ROS), and recruits caspases, thereby inducing apoptosis (67). In bladder cancer cells, ERIANIN upregulated the expression of BIM and BAD, and downregulated the expression of BCL-2; the IC_50_ value was 65.04 nM after treatment with ERIANIN for 48 h, indicating that ERIANIN activated the mitochondria-mediated apoptotic pathway by regulating the balance between pro-apoptotic and antiapoptotic protein expression (33).

**Figure 2:**
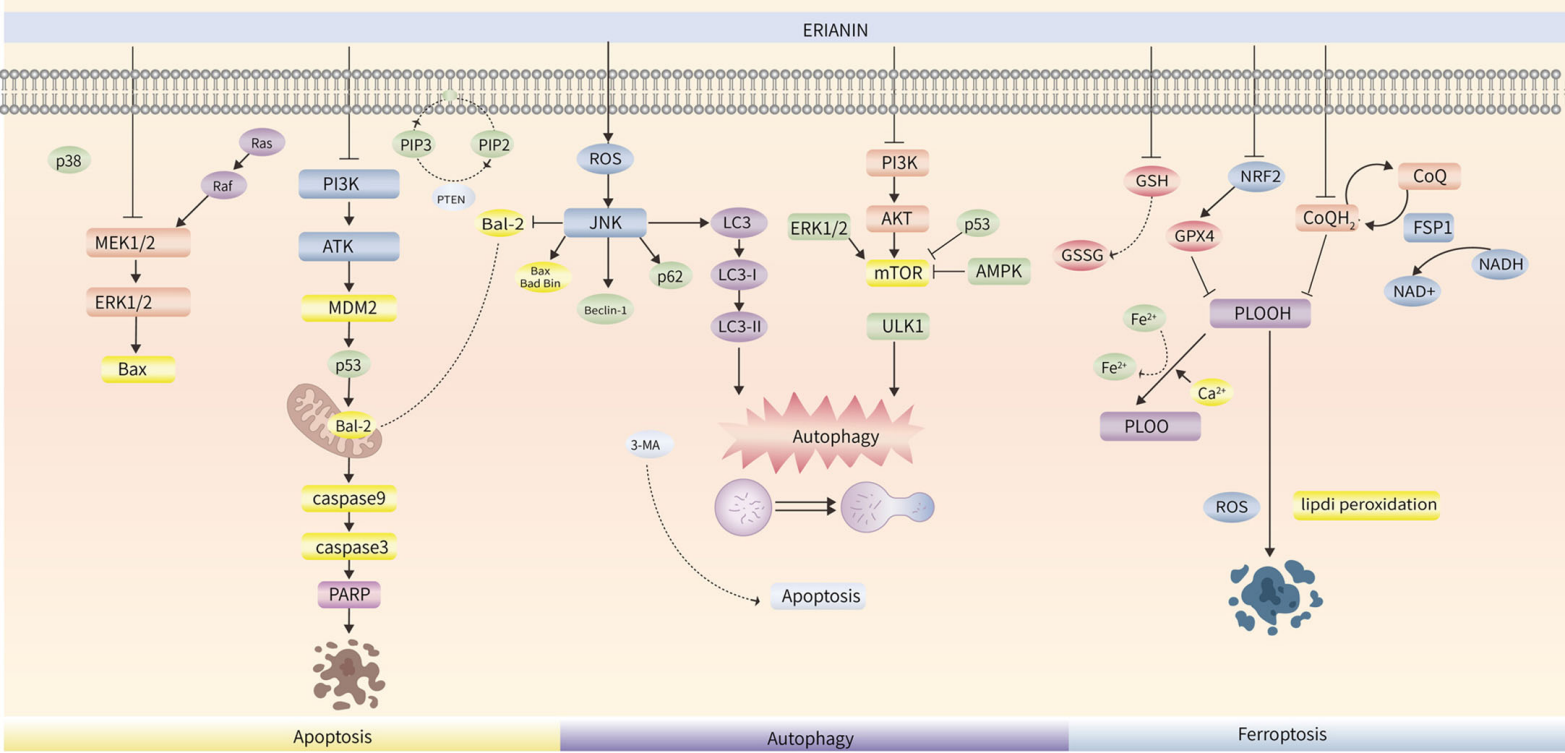
The role of ERIANIN in cell apoptosis, cell autophagy and Ferroptosis. ERIANIN regulates cell apoptosis through the MEK/ERK pathway and the PI3K/AKT pathway. ERIANIN regulates autophagy through the classical JNK/LC3 pathway and the PI3K/AKT/mTOR pathway; In addition, ERK, p53 and AMPK pathway also play a role in the process of autophagy through the mTOR pathway; ERIANIN regulates Ferroptosis through the NRF2/GPX4/PLOOH pathway and the CoQH2/PLOOH pathway. p38, p38 kinase; MEK1/2, serine/threonine protein kinase 1/2; ERK1/2, Extracellular signal-regulated kinase 1/2; Bax, BCL2-associated X protein; Ras, RAS oncogene homolog; Raf, Raf oncogene; PI3K, phosphatidylinositol 3-kinase; AKT, serine/threonine kinase; MDM2, MDM2 proto-oncogene; PARP, poly(ADP-ribose) polymerase; JNK, c-Jun NH2-terminal kinase; Bcl2, BCL2 apoptosis regulator; LC3, microtubule associated protein 1 light chain 3; AMPK, AMP-activated protein kinase; NRF2, NFE2 like bZIP transcription factor 2; GPX4, glutathione peroxidase 4; GSH, pyrimidodiazepine synthase; ROS, reactive oxygen species; PLOOH, phospholipid hydroperoxides; NAD+, nicotinamide adenine dinucleotide.

ERIANIN upregulated p-JNK, p-c-Jun, and p-BCL-2 (Ser70) to activate JNK signalling, and the JNK inhibitor SP600125 effectively inhibited ERIANIN-induced apoptosis (37). ROS is an important signal of apoptosis, and high levels of ROS activate the mitochondrial apoptotic pathway and increase the levels of JNK and c-Jun phosphorylation (68). ERIANIN induced an increase in ROS levels and activated the JNK/c-Jun pathway. N-acetyl cysteine (NAC) pre-treatment significantly reversed the inhibitory effects of ERIANIN on proliferation, apoptosis in osteosarcoma cells (37), suggesting that JNK activation is dependent on ROS and is an important pathway for ERIANIN-induced apoptosis. By causing ERS to upregulate the phosphorylation of IRE1a and eIF2a, ERIANIN interferes with endoplasmic reticulum homeostasis, increases BAX and CHOP protein expression levels, and promotes apoptosis in prostate tumor cells (32). In conclusion, ERIANIN induces tumor cell apoptosis, as shown in (**Figure 2**).

### 3.2 Autophagy effects

Autophagy is a fundamental form of degradation and recirculation of cytoplasmic components that is essential for maintaining cell survival and homeostasis (69). Autophagy mainly involves wrapping damaged organelles, error proteins, and microorganisms within vesicles through autophagosomes, interfusing with lysosomes, and hydrolysing vesicle contents to generate energy and nutrients for cellular reuse (70). Autophagy occurs upstream of apoptosis, and excessive activation of autophagy leads to apoptosis. Only a portion of chromatin is condensed in the death mode of autophagy, however, DNA fragments can be found in apoptotic cells (71, 72). Autophagy is a complex process that plays a dual role in tumor therapy, protecting cell survival and promoting cell death. LC3 is a marker of autophagy, and the autophagy junction protein p62 is a selective substrate for autophagy (73, 74). After treating osteosarcoma cells with ERIANIN, it was found that the intracellular acid-vesicle organelles increased, the expression of LC3-ll, p62, and Beclin1 was upregulated, and ROS accumulation was induced, which induced an increase in the phosphorylation levels of JNK and c-Jun, suggesting that ERIANIN activated the ROS/JNK pathway to induce autophagy in osteosarcoma cells. After using the autophagy inhibitor 3-MA, apoptosis was enhanced, suggesting that the autophagy-promoting effect of ERIANIN on osteosarcoma cells has a protective effect (37). Similar results were obtained in a study of ERIANIN in oral squamous cell carcinoma (75). In the ERIANIN-treated cells, green fluorescent autophagic vesicles were distinctly observed, the expression of LC3-I/LC3-II increased, and p62 decreased, indicating that autophagy was accompanied by apoptosis. The mitogen-activated protein kinase (MAPK) pathway is a crucial component of cellular signalling processes and regulates important intracellular functions, including proliferation, differentiation, migration, apoptosis, and autophagy. MAPK mainly includes ERK, JNK, and p38 MAPK. The phosphorylation levels of ERK1/2 and JNK1/2 were significantly increased after ERIANIN treatment, while the levels of p-p38 were decreased, but those of p-AKT were not significantly changed. The use of autophagy inhibitors revealed a significant decrease in ERK1/2 and JNK1/2 inhibitor-mediated autophagy but no significant change in p38, and the inhibition of autophagy increased the rate of apoptosis in both SAS and SCC9 cells. Since autophagy is involved in cell survival and death, the relative contribution of ERIANIN to cytotoxicity was assessed. ERIANIN induces apoptosis and autophagy by regulating the MAPK signalling pathway, which may be associated with cell survival. ERIANIN induces autophagy, as shown in **Figure 2** (**Figure 2**).

### 3.3 Ferroptosis effects

Ferroptosis is a new form of programmed cell death that is iron ion dependent, mainly manifested by an increase in ROS levels and the accumulation of lipid peroxides (76–78). Morphology is characterised by a decrease in mitochondrial volume, rupture of the outer membrane, increase in the density of the inner membrane, and decrease or disappearance of cristae without significant changes in the nucleus (76). The essence of this process is the alteration of plasma membrane fluidity and permeability. There are two main types of signalling pathways for ferroptosis: the GPX4/glutathione (GSH) pathway and the FSP1/CoQ10/NADH pathway. GPX4 is the only enzyme responsible for scavenging lipid peroxides in cells, and it plays an important role in maintaining lipid bilayer homeostasis in the cell membrane (77). Inhibition of GPX4 directly induces ferroptosis. FSP1 is a glutathione-independent inhibitor of ferroptosis that acts as an oxidoreductase and reduces coenzyme Q (CoQ) to panthenol (CoQH2) on the cell membrane. CoQH2 is a lipophilic antioxidant that traps free radicals and inhibits lipid peroxidation (79). The important products of ferroptosis are ROS and lipid peroxides.

Recent studies have found that ferroptosis is associated with the development of various malignancies, such as triple-negative breast cancer (80), liver cancer (81), lung cancer (82), and pancreatic cancer (83). Ferroptosis inducers can effectively kill a variety of tumor cells, exert antitumor activity, and have great development potential (84). Ca^2+^/CaM signaling is a key target for ERIANIN treatment, and the CaM protein is the main downstream molecule of the calcium signalling pathway. ERIANIN, a novel inducer of ferroptosis, exerts anticancer activity by activating Ca^2+^/CaM signalling to increase Ca^2+^ and Fe^2+^ levels, inducing ferroptosis and inhibiting cell migration in lung cancer cells (31). Lung cancer cells treated with ERIANIN showed a significant increase in ROS accumulation, GSH depletion, and lipid peroxidation, which are key events in ferroptosis. Mitochondrial matrix cohesion and expanded cristae formation were also observed. NAC and the ferroptosis inhibitors Ferrostatin-1 and Liproxstatin-1 attenuated ERIANIN-induced cell death. In addition, ERIANIN induces ferroptosis and lipid peroxidation in bladder cancer cells. After pre-treatment with shNRF2, GSH levels decreased significantly, but the activities of ROS and malondialdehyde greatly increased (85). It has been suggested that inactivation of nuclear factor e2-related factor 2 (NRF2) is the key factor for ERIANIN triggering ferroptosis in bladder cancer cells. Overall, ERIANIN promoted ferroptosis in tumor cells through the GPX4 and CoQ pathways, as shown in (**Figure 2**).

### 3.4 Cell cycle effects

The cell cycle is ubiquitous in living organisms, and malignant tumor cells are characterised by unlimited proliferation; therefore, inhibiting tumor cell proliferation is one way to treat tumors (86). Cell cycle processes involve the regulation of a variety of signals, such as cyclin-CDK-CKI, a signal regulatory network in the cell cycle, and the core regulatory protein is cyclin-dependent kinase (CDK) (87). CDKs need to bind to cyclin to form complexes to play the role of maintaining the cell cycle, and inhibition of CDK expression has become a means of treating tumors (35). p21 can inhibit CDK and is involved in a variety of cellular functions, including cell proliferation, damage, and apoptosis (88). ERIANIN inhibits the cell cycle by inducing cell arrest in the G2/M phase. Treatment of osteosarcoma cells with ERIANIN significantly increased the number of cells in the G2/M phase, significantly decreased those in the G0/G1 and S phases, upregulated the expression of p53, p21, p27, p-CDK1, and cyclin B1, and downregulated the expression levels of CDK1 and CDK7, indicating that ERIANIN inhibited the activation of the cyclin-CDK complex and caused cellular a G2/M block, thereby exerting cytotoxic effects (75). In a study on gastric precancerous lesions, ERIANIN decreased the protein expression levels of HRAS, AKT, and p-AKT, upregulated the expression level of the p21 protein, blocked the cell cycle, and caused cell arrest in the G2/M phase (24). In studies of liver cancer (26), lung cancer (89), and leukaemia diseases (40), ERIANIN similarly reduced cell viability, induced cell arrest in the G2/M phase, and inhibited cancer cell proliferation, which is consistent with previous studies.

**Figure 3:**
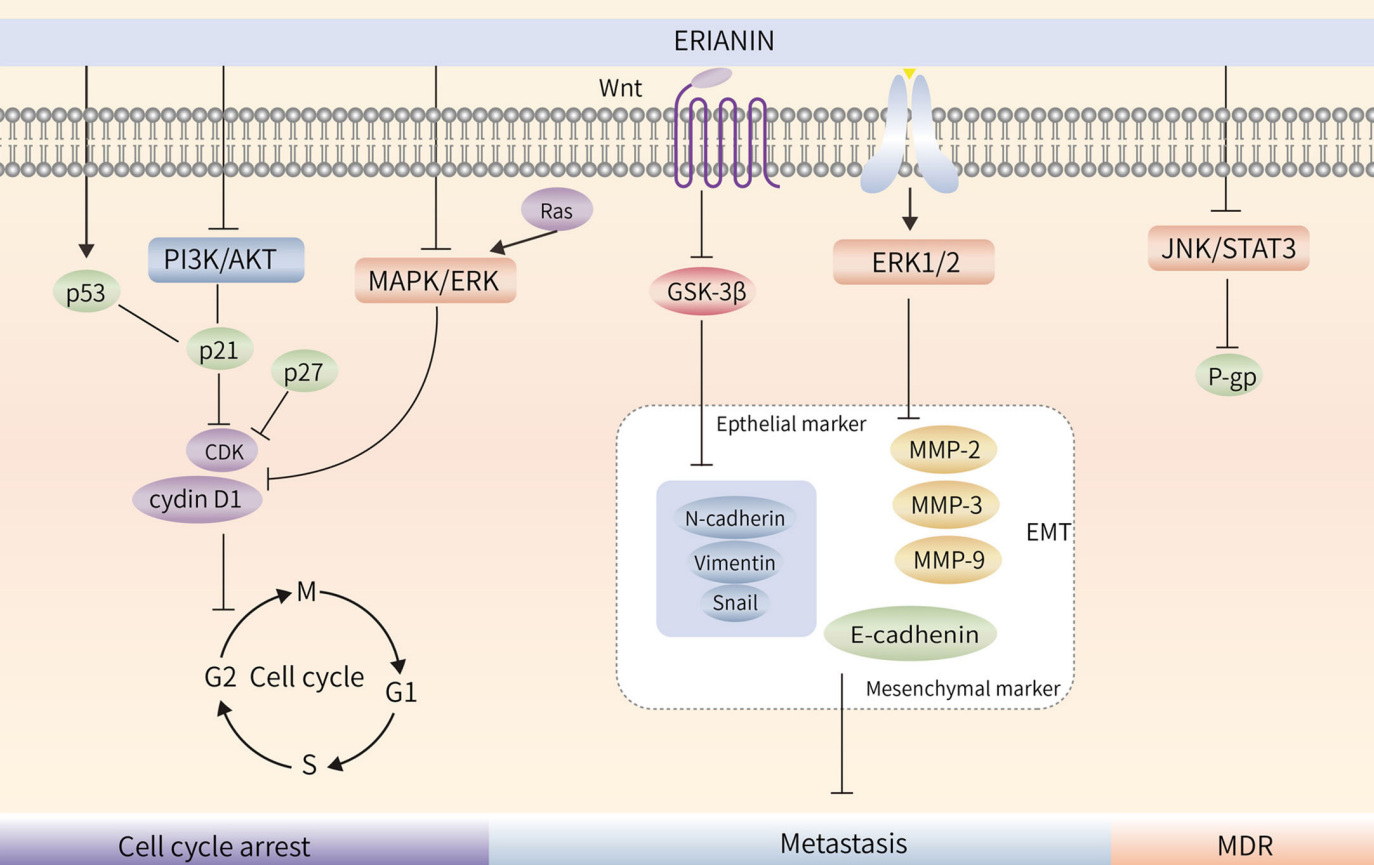
ERIANIN regulates cell cycle, metastasis and MDR. ERIANIN regulates cell cycle through the PI3K/AKT and MAPK/ERK pathway. ERIANIN regulates the EMT process through the GSK3β and ERK1/2 pathway. ERIANIN regulates MDR through the JNK/STAT3 pathway. PI3K, phosphatidylinositol 3-kinase; AKT, serine/threonine kinase; p21, cyclin-dependent kinase inhibitor 1A; p27, cyclin-dependent kinase inhibitor 1B; CDK, Cyclin-dependent kinase; MAPK, mitogen activated kinase-like protein; ERK, Extracellular signal-regulated kinase; Wnt, wingless-type MMTV integration site family; GSK3β, glycogen synthase kinase 3 beta; MMP2, matrix metallopeptidase 2; MMP3, matrix metallopeptidase 3; MMP9, matrix metallopeptidase 9; EMT, epithelial-mesenchymal transition; JNK, c-Jun NH2-terminal kinase; STAT3, signal transducer and activator of transcription 3; MDR, Multiple Drug Resistance; P-gp, ATP binding cassette subfamily B member 1.

Treatment of bladder cancer 5637 cells with ERIANIN resulted in a decrease in the number of cells in the G1 phase and an increase in those in the S and G2 phases; treatment also upregulated p21 protein expression and decreased p-AKT and AKT protein expression, indicating that ERIANIN inhibited bladder cancer cell proliferation through the PI3K/Akt pathway and blocked the cells in the G1 phase (90). ERIANIN inhibited RAS protein expression and the Raf/MEK/MAPK-ERK signalling pathway, decreased cellular cyclin D1 and ICAM-1 expression, promoted PD-L1 protein hydrolysis, and enhanced the killing effect of T cells in tumors (19). Similarly, ERIANIN blocked nasopharyngeal carcinoma cells in the G0/G1 phase (43). Thus, it is evident that the molecular mechanisms of ERIANIN-induced cell cycle blockade differ in different types of tumor cells. The blocking effect of ERIANIN on the cell cycle is shown in (**Figure 3**).

### 3.5 Metastasis effects

Local invasion and distant metastasis are the most important features of malignancy (91). Epithelial-mesenchymal transition (EMT) refers to the biological process of transforming epithelial cells into mesenchymal cells, in which epithelial cells lose their polarity, intercellular tight junctions, and adhesion junctions and acquire the ability to infiltrate and migrate, while possessing the morphology and characteristics of mesenchymal cells (92–94). When EMT occurs in tumor cells, the ability to migrate and invade is enhanced, thereby promoting distant metastasis of tumors. Matrix metalloprotein-2 (MMP-2) and Matrix metalloprotein-9 (MMP-9) are markers of tumor invasion and metastasis (95). After treatment of lung cancer cells, the expression of MMP-2 and MMP-9 was significantly reduced, the expression of mesenchymal markers Vimentin, N-cadherin, Slug, Snail, and MMP-9 was decreased, and the expression of epithelial marker E-cadherin was upregulated, indicating that ERIANIN inhibited the migration of lung cancer cells by inhibiting EMT (31). Similarly, in SMMC-7721 cells, ERIANIN inhibits the invasion of hepatoma cells by downregulating the expression of N-cadherin, MMP-2, and MMP-9 and upregulating the expression of E-cadherin (64).

ERIANIN regulates the Wnt/β-catenin pathway. Studies have shown that ERIANIN inhibits tumor cell invasion by reducing the expression of key downstream targets, c-myc and p-GSK-3β (Ser9), while reducing the expression levels of Vimentin and Snail (96). The steady-state expression of MPP and its suppressor TIMP is essential for breast cancer cell migration. By downregulating the activity of ERK1/2, ERIANIN inhibited the gene expression of MPP-2, MPP-9, and CDH2 and promoted the expression of the corresponding inhibitors TIMP1, TIMP2, and CDH1, thereby inhibiting the degradation of the extracellular matrix (35). Meanwhile, ERIANIN upregulated E-cadherin and downregulated N-cadherin and fibronectin expression to inhibit the migratory ability of cells (97). In summary, ERIANIN acts on the Wnt and ERK1/2 pathways and their downstream genes, MMP-2 and MMP-9, to inhibit tumor metastasis, as shown in (**Figure 3**).

### 3.6 Tumor multidrug resistance and metabolism

**Figure 4:**
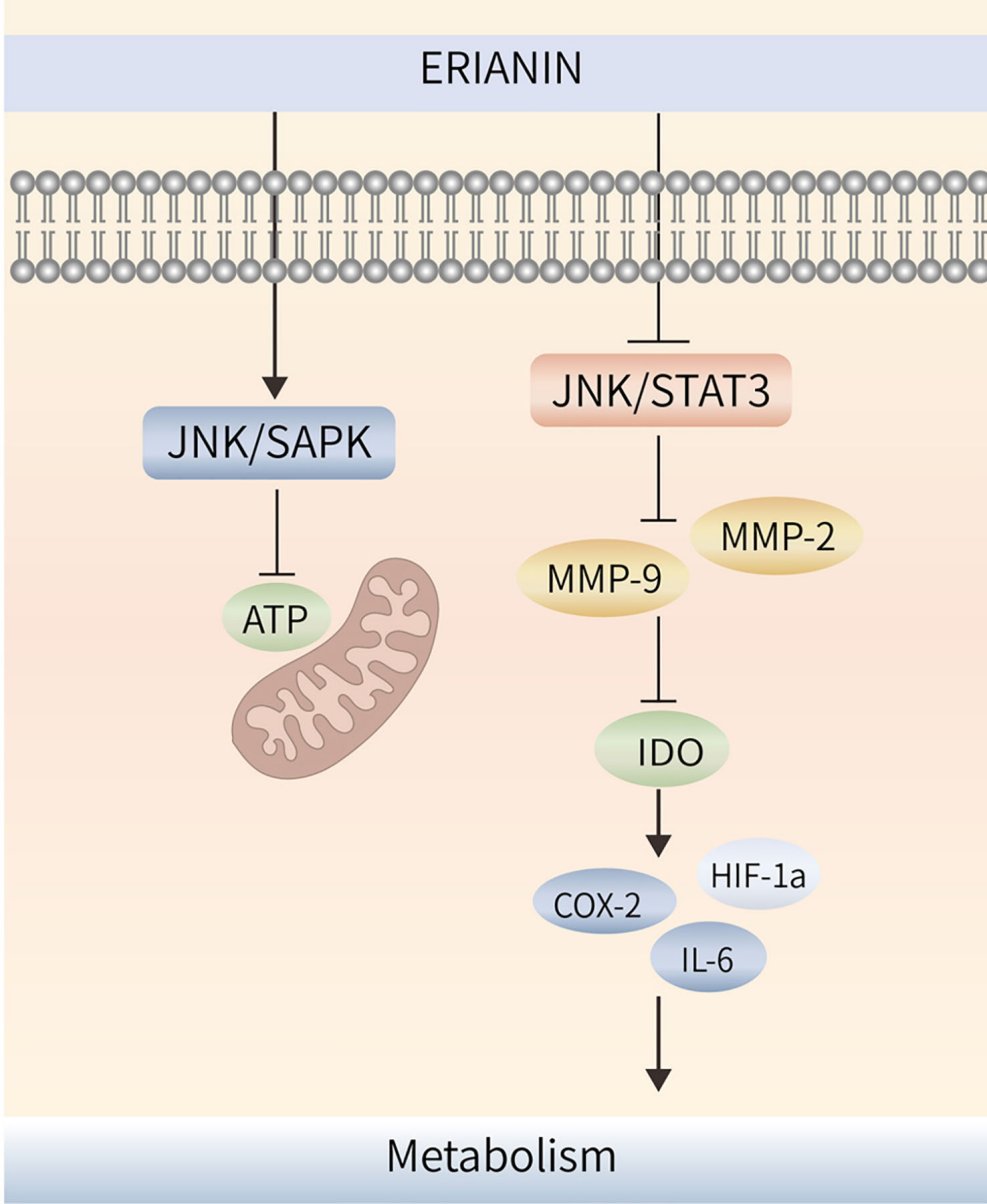
ERIANIN regulates cell metabolism through the JNK/SAPK/STAT3 pathway. ERIANIN induces activation of the JNK/SAPK pathway, downregulates the rate of extracellular acidification, reduces lactate production and glucose consumption, decreases cell survival, and reduces intracellular ATP content. In addition, ERIANIN can regulate the JAK/STAT3 pathway, inhibit the expression of p-JAK and p-STAT3, downregulate MMP-9 and MMP-2, reduce IDO activity, decrease the expression of IDO-mediated inflammatory mediators, including COX-2, HIF-1α, and IL-6. ATP, adenosine triphosphate; SAPK, mitogen-activated protein kinase 9; IDO, indoleamine 2,3-dioxygenase; COX-2, cytochrome c oxidase subunit II; IL-6, interleukin 6; HIF-1α, hypoxia inducible factor 1 subunit alpha.

Tumor multidrug resistance (MDR) is a phenomenon in which malignant tumor cells develop resistance to one antitumor drug along with resistance to multiple other antitumor drugs with different structures and mechanisms of action (98). MDR is the major cause of chemotherapy failure in colon cancer, and P-gp is encoded by the drug resistance gene MDR-1 (99). High P-gp expression is one of the main obstacles to therapeutic effects in colon cancer (100). ERIANIN can inhibit the activation of the JAK2/STAT3 signalling pathway, reduce P-gp expression, modulate the MDR phenotype of oxaliplatin-resistant cells, inhibit the proliferation of drug-resistant cells, and reverse oxaliplatin (LOHP) resistance (101) (**Figure 4**).

In addition, the abnormal proliferation of blood vessels around tumor tissue, which provides oxygen and nutrients to the tumor, plays an important role in tumor growth, invasion, and metastasis (102). Neovascularization is a marker of tumor progression, and the inhibition of angiogenesis is an important strategy for tumor treatment (103). Oxidative phosphorylation that occurs during mitochondrial respiration is essential for maintaining high levels of ATP, which are determinants of cell function and death (104, 105). However, glycolysis is an important pathway for the endothelial cell energy supply (106).

ERIANIN regulates angiogenesis through the cellular metabolic process. ERIANIN induces activation of the JNK/SAPK pathway, downregulates the rate of extracellular acidification, reduces lactate production and glucose consumption, decreases cell survival, and reduces intracellular ATP content, which causes mitochondrial dysfunction and inhibits the metabolism of human umbilical vein endothelial cells, suggesting that ERIANIN has antiangiogenic effects (107). The antitumor activity of ERIANIN was more effective against melanoma A375 than against hepatocellular carcinoma Bel7402, which may be due to the more abundant vascular distribution ratio of A375 tumors, where ERIANIN significantly caused vascular closure and inhibited neovascularisation (44).

Indoleamine 2,3-dioxygenase (IDO) has the function of regulating tumor angiogenesis (108). As shown in **Figure 4**, ERIANIN can regulate the JAK/STAT3 pathway, inhibit the expression of p-JAK and p-STAT3, downregulate MMP-9 and MMP-2, reduce IDO activity, decrease the expression of IDO-mediated inflammatory mediators (COX-2, HIF-1α, and IL-6), significantly inhibit the metastatic ability of lung cancer cells, block the angiogenic mimicry of 2LL-IDO cells, disrupt the tubular structure of human umbilical vein endothelial cells, and inhibit the proliferation of vascular endothelial cells (109, 110). This suggests that ERIANIN can regulate cell metabolism and is an effective vascular blocker that can inhibit angiogenesis and prevent malignant tumors by inhibiting IDO expression (**Figure 4**). ZJU-6 is a novel ERIANIN derivative with strong antiangiogenic and free radical-scavenging abilities, which can enhance the antitumor ability of ERIANIN (111).

## 4 Significances of ERIANIN in innate immunity

### 4.1 Anti-inflammatory effects

**Figure 5:**
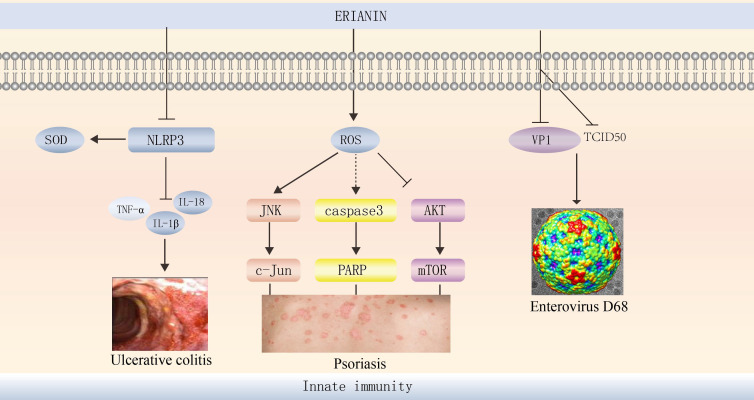
ERIANIN regulates innate immunity. ERIANIN is a specific inhibitor of NLRP3 activation *in vitro*, reducing IL-1b, TNF-α, and IL-18 levels, up-regulating SOD levels. ERIANIN inhibits cell proliferation through the AKT/mTOR pathway and induces cell apoptosis through the ROS-mediated JNK/c-Jun and caspase3/PARP signalling pathways and then regulates the psoriasis. ERIANIN has a protective effect against EVD68-induced cell damage by inhibiting the production of human intestinal EVD68 virus by decreasing the expression of VP1 and TCID50. NLRP3, NLR family pyrin domain containing 3; SOD, superoxide dismutase; IL-18, interleukin 18; IL-1b, interleukin 1b; TNF-α, tumor necrosis factor alpha; ROS, reactive oxygen species; PARP, poly (ADP-ribose) polymerase; VP1, capsid protein 1; TCID50, tissue culture infective dose 50%.

ERIANIN exerts anti-inflammatory effects in diseases such as joint swelling and ulcerative colitis (UC). ERIANIN is a specific inhibitor of NLRP3 activation *in vitro*, reducing IL-1b and IL-18 levels, decreasing neutrophil migration, selectively inhibiting NLRP3 inflammatory vesicle formation, and suppressing MSU-induced acute joint swelling (112). In a UC mouse model, ERIANIN significantly increased superoxide dismutase levels in serum and colonic tissue, reduced ROS accumulation, decreased neutrophil and monocyte counts, reduced IL-1β, IL-6, and TNF-α levels, and reduced cellular peroxidative damage and immune inflammatory responses by modulating inflammation and oxidative stress levels (113). ERIANIN may act on the host by inhibiting inflammatory pathways, enhancing immunity, interfering with several bacterial virulence factors, and reducing mortality in *S. aureus*-infected mice (114) (**Figure 5**).

### 4.2 Antiviral effects

Human enterovirus 68 (EVD68) is a major pathogen of respiratory diseases worldwide (115), and ERIANIN has a protective effect against EVD68-induced cell damage by inhibiting the production of human intestinal EVD68 virus by decreasing the expression of VP1 and TCID50 (116) (**Figure 5**). Thus, ERIANIN is a potential drug for the treatment of human enterovirus.

### 4.3 The role of ERIANIN in psoriasis

Psoriasis is a chronic inflammatory skin disease characterised by hyperproliferation and abnormal keratinocyte differentiation (117). ERIANIN can reduce mitochondrial membrane potential and increase cytoplasmic calcium levels. It exerts antiproliferative and pro-apoptotic effects on human immortalised keratinocytes (HaCaTs) through mitochondrial pathways and ERS (118) and significantly reduces cell viability for treating psoriasis (119). ERIANIN inhibits HaCaT proliferation and induces HaCaT apoptosis through the ROS-mediated JNK/c-Jun and AKT/mTOR signalling pathways (120).

## 5 Other pharmacological effects

ERIANIN inhibits neovascularization and delays the development of diabetic retinopathy (121). The Rho/ROCK signalling pathway is involved in the regulation of angiogenesis and permeability. ERIANIN selectively inhibits collagen-mediated neovascularization, interferes with collagen-cell interactions, and suppresses human retinal vascular endothelial cells by inhibiting the activity of the RhoA/ROCK1 pathway and decreasing integrin cell proliferation, thereby inhibiting retinal angiogenesis (121). This suggests that ERIANIN also has therapeutic potential for collagen-mediated retinal angiogenesis.

Furthermore, ERIANIN has protective effects against high glucose-induced oxidative damage in renal tubular epithelial cells (122). Sustained hyperglycaemic stimulation induces apoptosis in NRK-52E cells, and ERIANIN acts as an original therapeutic target for diabetic nephropathy by blocking the ROS/MAPK/NF-κB signalling pathway, inhibiting ROS and malondialdehyde production, increasing the ratio of GSH to glutathione disulfide, alleviating oxidative stress, and inhibiting hyperglycaemia-induced renal insufficiency (122). In diabetic retinopathy, ERIANIN reduces cellular glucose uptake, inhibits downstream ERK1/2-NFκB pathway activation (123), decreases the expression of VEGF receptor 2 and its downstream signals, inhibits VEGF-induced angiogenesis (124), and attenuates microglia-induced retinal inflammation, thereby alleviating diabetic retinopathy.

## 6 Conclusion and future perspectives

Although great progress has been made in the treatment of cancer, it is still a high-mortality disease, and the trend of an ageing society has increased the risk of tumor diseases (125, 126). Tumor-targeted drugs have the characteristics of strong specificity and good tolerability, occupying an increasingly important position in tumor therapy, and screening of low-toxicity, safe, and effective antitumor drugs from Chinese herbal extracts has become a focus of modern antitumor drug research (127).

ERIANIN is a natural compound derived from Chinese herbal medicine with a unique molecular structure and significant pharmacological effects. It has promising therapeutic effects in tumors, inflammation, psoriasis, diabetic nephropathy, and retinopathy. Moreover, it has broad potential in antitumor therapy by promoting tumor cell apoptosis, autophagy, and ferroptosis, as well as blocking the cell cycle and inhibiting cell migration and neovascularisation through the multiple signaling pathways. However, given that the feedback loops in signaling pathways are widely known, it is crucial to consider how ERIANIN may affect these feedback loops. How ERIANIN may influence feedback loops and how this may contribute to its antitumor effects. For example, ERIANIN decreased the expression of phosphorylated ERK1/2 and inhibited the ERK1/2 signaling pathway in gastric cancer, breast cancer, melanoma and colorectal cancer (24, 35, 43, 122, 128). ERIANIN induced an increase in ROS levels in psoriasis, high glucose-induced injury, lung cancer and osteosarcoma (29, 37, 120, 124). As we known, ROS can stimulate ERK signaling via the activation of upstream activators and inactivation of catalytic activity of DUSPs, which leads to sustained ERK activation due to the lack of negative feedback responses elicited by dual-specificity phosphatases in many cancers (129, 130). In addition, a lot of literature reports that ferroptosis is governed by the efficiency of reactive oxygen species (ROS) production through the ERK pathway in cancer cells (131, 132). Dragana Savic et al. found that as ferroptosis induction via erastin is strongly dependent on the expression of Erk1/2 associated with the modulation of the ratio between ROS production and expression of ROS scavengers, phosphorylated Erk1/2 can be used as a predictor for cancer cells’ responses to erastin (133). More importantly, Yu et al. also reported that erianin could promote the accumulation of lethal lipid-based reactive oxygen species (ROS) and the depletion of glutathione (GSH) through NRF2, suggesting the induction of ferroptosis (85). In summary, these regulated signaling pathway networks including the Erk1/2 pathway are complex in the tumor microenvironment. ERIANIN plays an antitumor role by regulating these complex feedback loops in different cancers. Although numerous studies described in the present review have conducted substantial work on the mechanisms by which ERIANIN are involved in cancer signal transduction, research on the complex regulatory networks in the tumor microenvironment (TME) formed by multiple pathways are still lacking. Most of the research on ERIANIN focuses on cell lines and immunodeficient mice experiments, with few tumor immunological studies and a lack of clinical observation data in immune-complete mice and humans. Whether ERIANIN exerts antitumor effects and could increase sensitivity to immune checkpoint therapy (ICB) in “cold” solid tumors is unknown. Whether ERIANIN can improve the prognosis and prolong the survival of cancer patients is also unknown. There is still a lack of in-depth exploration of the direct targets, structural optimisation, and drug delivery process *in vivo* of ERIANIN in immune-complete mice and humans. In addition, moving from basic experiments to clinical research should be the focus for the future, and is also an inevitable requirement for clinical practice.

In summary, this article reviewed the existing effects of ERIANIN and elaborated on the molecular mechanism of its antitumor activity, thereby providing a reference for future research on traditional Chinese medicines. ERIANIN-targeted therapy may be a novel and potentially effective strategy for cancer patients. Thus, further understanding of the ERIANIN network, especially in the area of cancer immunotherapy, is required.

## Author contributions

ZY, RXL, MHQ, HWM, MG and HQW collected the related studies and drafted the manuscript. HQW, MG and ZY participated in the design of the review. ZY, JH, HQW, TS, DDZ and KZ and MG initiated the study and revised the manuscript. All authors have read and agreed on the published version of the manuscript.
